# Antibacterial Cu-doped cotton textile against respiratory pathogens for preventing hospital-acquired infections

**DOI:** 10.3389/fbioe.2025.1641123

**Published:** 2025-09-10

**Authors:** Jianhui Gao, Deliang Liu, Zhiqiang Lin, Yang Zhou, Zhuojun He, Xiafei Dai, Pengfei Zhao, Hongzhou Lu, Mingbin Zheng

**Affiliations:** ^1^ National Clinical Research Center for Infectious Disease, Shenzhen Third People’s Hospital, Southern University of Science and Technology, Shenzhen, China; ^2^ The Affiliated Dongguan Songshan Lake Central Hospital, Guangdong Medical University, Dongguan, China

**Keywords:** functional textile, antibacterial effects, Cu-O coordination bonds, reactive oxygen species, antibacterial Cu-doped cotton textile

## Abstract

Respiratory pathogens transmitted *via* clinical textiles represent a major source of hospital-acquired infections, yet current antibacterial fabric strategies are limited by poor durability and weak bacterial inhibition. Here, we reported a molecular-level strategy for the fabrication of copper-doped antibacterial cotton textiles (Cu@textile) *via* a simple immersion of common cotton in a Cu(II)-saturated NaOH solution. This process enabled stable coordination between the copper ions and cellulose hydroxyl groups, forming stable Cu-O bonds throughout the fiber matrix. Structural and spectroscopic analyses confirmed uniform copper integration and chemical bonding. The resulting Cu@textile exhibited potent, broad-spectrum antibacterial activity against key respiratory pathogens, including *Pseudomonas aeruginosa*, *Acinetobacter baumannii*, and *Mycobacterium tuberculosis*, with >99% sterilization efficiency. Mechanistic studies revealed this efficacy as copper-induced reactive oxygen species (ROS) production and bacterial membrane disruption. This accessible and scalable antimicrobial textile eliminates the need for specialized equipment or nanoparticle synthesis, and may represent a strategic intervention to reduce bacteria propagation and contact infection risks in healthcare settings.

## 1 Introduction

Hospital-acquired infections (HAIs) is a profound and persistent threat to global healthcare, affecting millions and placing immense strain on the overburdened medical system (Thorpe et al., 2024; Laco et al., 2025; Hema et al., 2025). Respiratory pathogens causing HAIs can spread through human activities, with clinical textiles (e.g., bed sheets, medical drapes, and patient attire) serving as main vectors for bacterial proliferation and transfer (Nieto-Rosado et al., 2024; Lionakis and Hohl, 2020; Tang et al., 2022; Hasanzadeh et al., 2023). In the past few years, *various approaches* have been investigated to incorporate antibacterial properties with cotton textiles. Antimicrobial additives such as organic compounds, synthetic or natural polymers, and metal-based materials have been incorporated into textiles through physical methodologies (Fu et al., 2021; Das et al., 2025; Qin et al., 2021; Feng et al., 2025; Zhang et al., 2024). However, these methods merely deposit antibacterial components onto the fabric surface without fully leveraging the material’s molecular structure. While antibacterial coatings can effectively inactivate bacteria attached to clinical textiles, they suffer from limitations in coating durability due to low adhesivity and weak binding (e.g., weak electrostatic interactions) (Zhang et al., 2022; Chen et al., 2024; Kocgozlu et al., 2024; Wang et al., 2023). Therefore, developing a novel strategy with stable adherence and bactericidal performance for designing antibacterial clinical textiles is crucial to prevent the spread of bacteria within hospital environments and combat HAIs.

To address this challenge, we proposed a straightforward molecular-level strategy to fabricate antimicrobial cotton textile. By immersing the cotton textile in a Cu(II)-saturated NaOH solution, copper ions infiltrate the cellulose matrix of the cotton textile and strongly coordinate with the oxygen atoms on the cellulose chains, thereby yielding Cu-doped cotton textiles (Cu@textile). The presence and distribution of copper on the Cu@textile surface were validated by scanning electron microscopy (SEM) and energy dispersive spectroscopy (EDS). Additionally, the chemical states of copper on the Cu@textile surface were further analyzed by X-ray diffraction (XRD) and X-ray photoelectron spectroscopy (XPS). The antibacterial mechanism and effect of Cu@textile against common respiratory pathogens (*Pseudomonas aeruginosa*, *Acinetobacter baumannii*, and *Mycobacterium tuberculosis*) in hospital environments were investigated. This methodology may represent a potent technology for the inactivation of various respiratory bacteria within hospital environments, and has great potential in preventing nosocomial infections.

## 2 Materials and methods

### 2.1 Materials

Sodium hydroxide (NaOH), isopropanol, 2.5% glutaraldehyde and ethanol were purchased from Shanghai Lingfeng Chemical Reagent Co., Ltd (CHN). Copper wires were purchased from Jinlongyu Group Co., Ltd (CHN). Cotton textiles were purchased from Guangzhou Dajia Biotechnology Co., Ltd (CHN). 2,7-dichlorofluoresin diacetate (DCFH-DA), LB broth, LB agar and glycerol were purchased from Sigma-Aldrich (United States). Difco Middlebrook 7H9 and Difco Middlebrook 7H10 were purchased from Becton Dickinson (United States). Oleic albumin dextrose catalase (OADC) was purchased from Bio-Rad Laboratories (United States). Glutathione (GSH), hydrogen peroxide solution (H_2_O_2_) and aqueous ammonia were purchased from Aladdin (CHN). Human embryonic kidney 293 (HEK-293) cells were purchased from the American Type Culture Collection (ATCC). Dulbecco’s modified eagle medium (DMEM), Fetal bovine serum (FBS), penicillin/streptomycin, trypsin-ethylenediaminetetraacetic acid (trypsin-EDTA), Phosphate-buffered saline (PBS) were obtained from Gibco (United States). L-histidine monohydrochloride monohydrate was purchased from Yuanye biotechnology Co., Ltd (CHN). NaCl was purchased from Solarbio (CHN). Na_2_HPO_4_·2H_2_O was purchased from Beyotime (CHN). Cell counting kit-8 (CCK-8) was purchased from Dojindo Laboratories (JPN). All aqueous solutions were prepared using ultrapure water.

### 2.2 Preparation of the Cu@textile

The composites used in this study were NaOH, Copper wires and Cotton textiles. Preparation of Cu-doped cotton textile included a previously reported protocol (Qian et al., 2022) with modifications. First, NaOH was dissolved in double distilled water (ddH_2_O) to obtain 10 wt% NaOH solution. Then, copper wires were immersed in NaOH solutions, which were maintained static until the blue color of solution stabilized (generally in 3 days), thus yielding the Cu(II)-saturated NaOH aqueous solution. Subsequently, the textile samples were soaked in the Cu(II)-saturated NaOH solutions until they displayed a uniform blue color (generally in 2 days). The Cu-doped cotton textile samples were then repeatedly rinsed with ddH_2_O, followed by air dry at room temperature.

### 2.3 Characterization of the Cu@textile

Thermogravimetric analysis (TGA) was conducted to determine the copper contents of the textiles. TGA analysis was performed using a TGA Q50 thermogravimetric analyzer (TA Instruments, United States) at a heating rate of 10 °C min^−1^ and a nitrogen flow of 50 mL/min. The surface morphology of textiles were analyzed using scanning electron microscopy (SEM). SEM images were taken using an APREO S SEM (Thermo Fisher Scientific, United States) at 5 kV accelerating voltage. Elemental mapping was obtained using energy dispersive spectroscopy (EDS) attached to the SEM. X-ray diffraction (XRD) was utilized to observe the crystalline structures of textile cellulose using LabX XRD-6100 diffractometer (SHIMADZU, JPN) with a copper X-ray tube (Ka, λ = 1.5418 Å) at 40 kV and 30 mA. The XRD data were analyzed by HighScore Plus software. The chemical state of copper ions on textiles were determined by X-ray photoelectron spectroscopy (XPS). For XPS analysis, a PHI 5000 Versaprobe III X-ray spectrometer (ULVAC PHI, JPN) was utilized and the data were analyzed by CasaXPS software.

### 2.4 Bacteria culturing

*P.aeruginosa* (ATCC 27853), *A. baumannii* (ATCC 19606) and *M. tuberculosis H37Ra* (ATCC 25177) were purchased from the American Type Culture Collection (ATCC, United States). *P*.*aeruginosa* and *A. baumannii* were cultured in LB medium. *Mycobacterium tuberculosis* was cultured in Middlebrook 7H9 liquid medium (50 mL) supplemented with 10% OADC and 0.5% glycerol. Bacteria were cultured at 37 °C with continuous shaking at 120 rpm.

### 2.5 ROS detection

The production of ROS was evaluated using a DCFH-DA fluorescence assay. Briefly, the Cu-doped cotton textile was incubated with 1 mM H_2_O_2_ at ambient temperature. After treatment, DCFH-DA was added to each well for 20 min. Then, the fluorescence intensity of the solutions was measured at an excitation wavelength of 488 nm using a Synergy Neo2 microplate reader (BioTek, United States). To investigate the mechanism of ROS-induced bacterial death, SEM images was uesd to observe the morphologies changes of *P*. *aeruginosa* after incubating with Cu@textile or untreated textiles. After 3 h incubation with bacteria, the textile samples were collected and washed with ddH_2_O to eleminate the adhered bacteria and culture medium. Then they were fixed in 2.5% glutaraldehyde for 12 h at 4 °C. Finally, the samples were dehydrated through a gradient ethanol series (30, 50, 70, 90 and 100 wt%) and lyophilized for the SEM.

### 2.6 Antibacterial assessment

The cytotoxicity of Cu@textile was assessed using the CCK-8 assay (Qian et al., 2022; Yu et al., 2024). HEK 293 cells were seeded and incubated for 24 h. Following ISO 12947–2:2016, artificial perspiration was prepared. A piece of the textile sample was added to artificial perspiration. After 3 h, the artificial perspiration extract was collected and added to HEK 293 cells. Medium was replaced with 10% CCK-8 in serum-free DMEM and measured cytotoxicity using Synergy Neo2 microplate reader (BioTek, United States). For the evaluation of antibacterial properties, textile samples were exposed to liquid bacterial cultures for 3 h. Textile samples (1.5 cm × 2.5 cm) were used for antibacterial assessment. Before experiment, the textile samples were subjected to ultrasonic sterilization in isopropanol for 5 min, followed by two rinses with ddH_2_O. *P*.*aeruginosa*, *A. baumannii* and *M. tuberculosis* suspensions were diluted to OD_600_ = 0.2 (absorbance at 600 nm). Then, 2 mL of diluted bacterial suspensions and textile samples were placed in a 12-well plate and incubated at 37 °C, 150 rpm for 3 h. After incubation, the bacterial cells were centrifuged at 8,500 rpm for 10 min and washed with PBS twice. Then, the bacteria suspension was serially diluted, and 4 μL of each serial dilution was plated in triplicate onto LB agar for *P. aeruginosa* and *A. baumannii*, and 7H10 agar for *M. tuberculosis*. *P*.*aeruginosa* and *A. baumannii* plates were cultured at 37 °C for 8h, and *M. tuberculosis* plates for 21 days. After incubation, the plates were visualized by automated colony counter (Hangzhou shineso technology Co., Ltd., CHN) and the number of colonies were counted manually. To confirm the ROS-dependent bacterial killing of Cu@textile, GSH was used as an antioxidant to conduct the scavenger experiment. All procedures were identical to the antibacterial assessment described above, except that the samples were divided into three groups: (i) textile + bacteria, (ii) Cu@textile + bacteria, and (iii) Cu@textile + bacteria in the presence of 50 mM GSH.

### 2.7 Mechanical property test and air permeability test

The washing test was used to test the Cu@textile mechanical property based on an international standard (ISO 6330–2012). A front-loading, horizontal-drum-type washing machine (Haier, CHN) was used. A piece of Cu@textile sample (1.5 cm × 2.5 cm) was loaded into the washing machine with sufficient test clothing and 20 g of detergent (ECE reference detergent 98). The washing time was 20 min. After washing, the Cu@textile sample was removed from the washing machine and dried at room temperature. The tensile properties of the samples were characterized using an Instron 68TM-30 tester (Instron, United States) with a 1,000 N load cell. The textile samples (1 cm × 5 cm) were uniaxially stretched at a strain rate of 0.5 mm min^−1^. At least five specimens were measured from each type of sample. The air permeability of the Cu@textile was assessed using a simple ammonia-vapour diffusion test (Yang et al., 2024). Specifically, a glass vial was charged with 5 mL of 25% (w/w) aqueous ammonia and sealed with a piece of the textile sample. A strip of pH-indicator paper was suspended above the fabric to record the time required for the indicator change from yellow to blue.

### 2.8 Statistical analysis

All experimental data were presented as means ± standard error of mean, with experiments repeated at least three times. The statistical analysis was performed using GraphPad Prism 8.0 software.

## 3 Results and discussion

### 3.1 Characterization of Cu@textile

**FIGURE 1:**
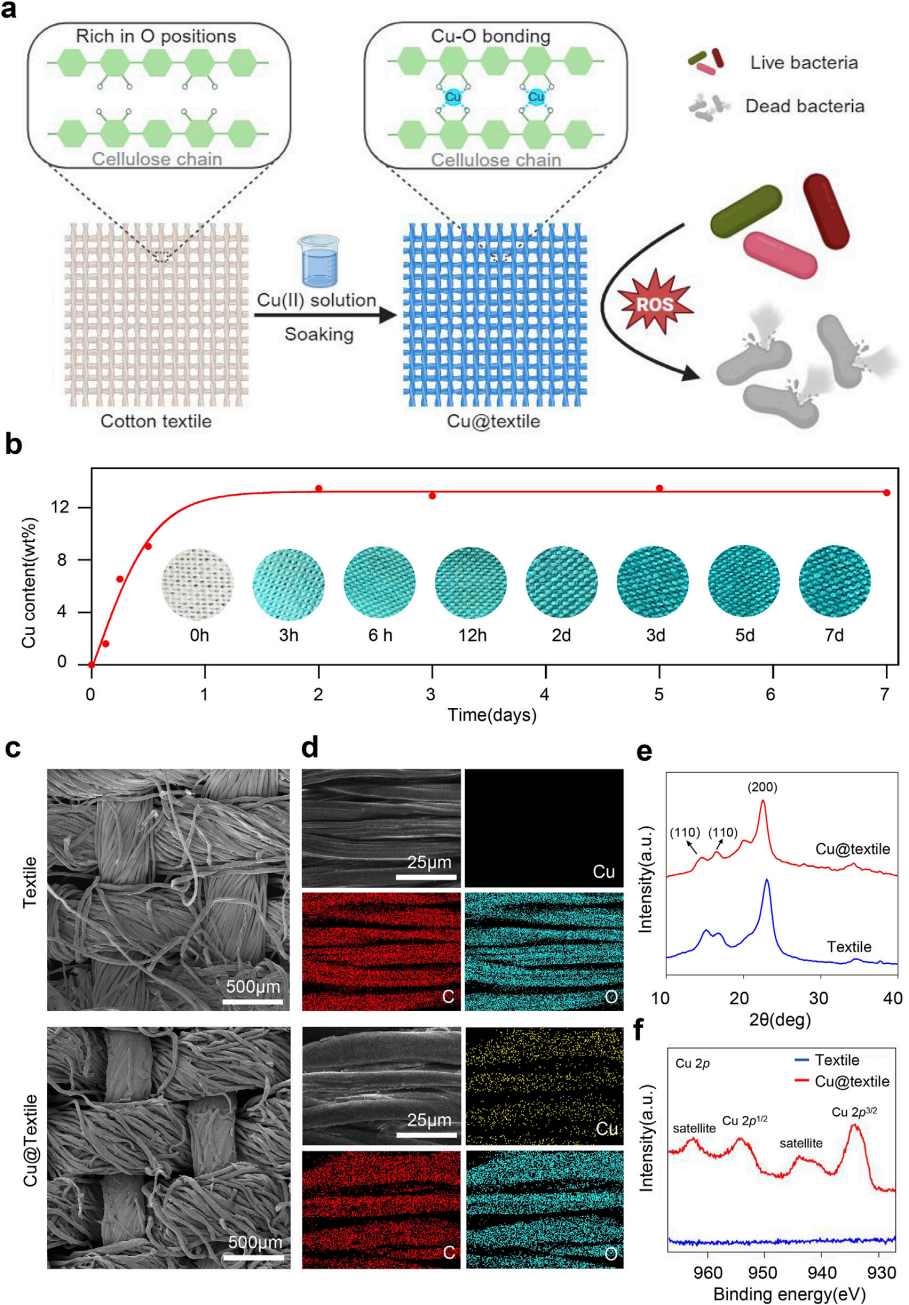
Preparation and characterization of the antibacterial Cu@textile. **(a)** Schematic illustration of the structure and broad-spectrum antibacterial effect of Cu@textile, achieved by soaking cotton textile in Cu(II)-saturated NaOH solution. During this process, copper ions coordinated with oxygen atoms on the textile cellulose chain to form Cu-O coordination bonds. Benefiting from reactive oxygen species (ROS) induced by copper ions, Cu@textile demonstrated efficient antibacterial activity. **(b)** Copper content on the cotton textile after different soaking times. The intense blue color represents high copper content. Insets: corresponding photographs of the textile samples. **(c, d)** SEM images of textile and Cu@textile with corresponding copper elemental mapping. **(e)** X-ray diffraction curve of textile and Cu@textile. **(f)** XPS Cu 2*p* spectrum of textile and Cu@textile.

Antibacterial Cu@textile was prepared by simply immersing cotton textiles into Cu(II)-saturated NaOH solution, where oxygen atoms in cellulose coordinated with copper ions to form robust Cu-O bonds (Figure 1a). Briefly, cotton textile was initially soaked in 10 wt% Cu(II)-saturated NaOH solution till no further color deepening occurred. The blue textile was subsequently taken out and rinsed with ddH_2_O. Finally, the textile was dried and prepared for use. To investigate the optimal soaking duration for Cu@textile preparation, we analyzed the copper content of Cu@textile at various soaking times using thermogravimetric analysis (TGA). The copper content of Cu@textile exhibited a gradual increase with the soaking time, and reached a plateau of 13.5 wt% after 2 days (Figure 1b). Hence, soaking textile in Cu(II)-saturated NaOH solution for 2 days was settled for Cu@textile preparation.

SEM images demonstrated that Cu@textile achieved abundant copper doping on the textile surface, and retained textile microstructure with no morphological change or fiber fracture (Figure 1c). Energy dispersive spectroscopy (EDS) further explored the distribution of copper on Cu@textile. As shown in Figure 1d, the distribution of copper on Cu@textile corresponded with that of carbon (C) and oxygen (O), indicating homogeneous distribution of copper throughout the cellulose microfibers. Quantitative element content statistics of Cu@textile were shown in Supplementary Figure S1, the relative standard deviation of the copper content was <3% across three batches, demonstrating both a uniform distribution and excellent batch-to-batch consistency. Furthermore, we employed X-ray diffraction (XRD) and X-ray photoelectron spectroscopy (XPS) to determine the chemical states of copper on Cu@textile surface. We firstly investigated the possible insertion mechanism of copper on Cu@textile. The crystal structure of cellulose remained unchanged before and after copper integration, evidenced by the similarity between the X-ray diffraction patterns of the control and Cu@textile (Figure 1e). Subsequently, the coordination state of copper ions was confirmed using X-ray photoelectron spectroscopy (XPS). The Cu 2*p* XPS spectrum of Cu@textile (Figure 1f) showed an apparent satellite peak at 943.7 eV and a Cu 2*p* ^3/2^ peak at 933.9 eV, indicating the presence of Cu (II) ions and the formation of Cu-O coordination bonds. Altogether, our approach demonstrated that after soaking in Cu (II)-saturated NaOH solution, Cu (II) ions were trapped into the cellulose crystals and the intercrystalline gaps, forming stabilized Cu@textile *via* coordination bonds.

As shown in Supplementary Figures S2a–d, No apparent changes of color or decreased integrity were observed for the Cu@textile sample after washing. The X-ray diffraction (XRD) profiles of the Cu@textile before and after washing were similar, indicating no structural changes. In terms of mechanical property, we performed the mechanical of the Cu@textile by an Instron tester. The tensile strength of Cu@textile was 34.30 MPa, which was ∼4.3% higher than that of the textile (32.88 MPa) (Supplementary Figures S2e, f). Additionally, the fracture area of the Cu@textile was compact, while that of the textile was loose. In terms of air permeability, we assessed the air permeability of the Cu@textile with a simple ammonia-vapor diffusion test. As shown in Supplementary Figure S2g, the Cu@textile allowed the ammonia to go through and discolor the pH test paper within 1 s, suggested the Cu@textile had excellent air permeability. In conclusion, we have confirmed that the fabric has excellent air permeability, mechanical properties and washing stability.

### 3.2 Antibacterial activity

**FIGURE 2:**
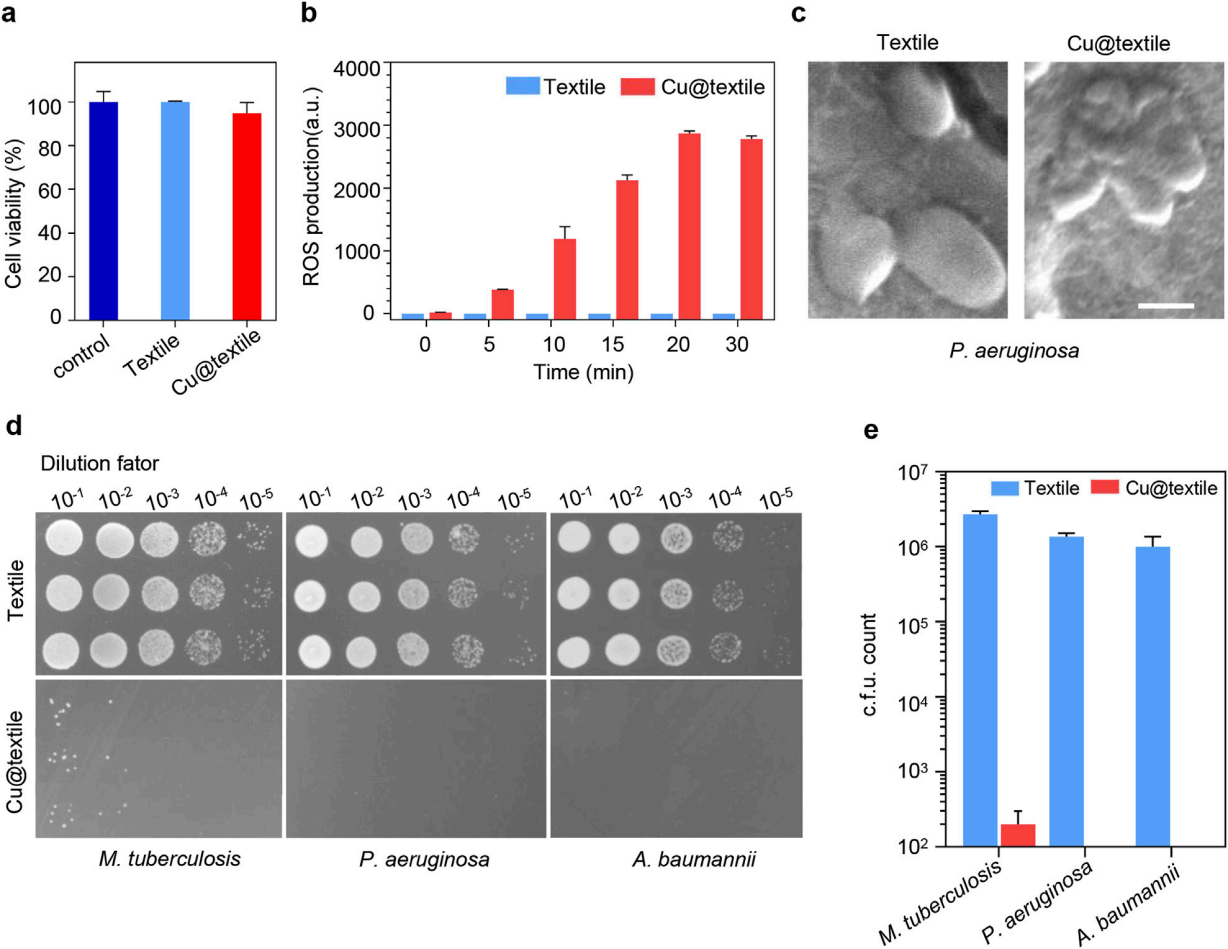
The antibacterial activity of Cu@textile. **(a)** Cytotoxicity of Cu@textile to HEK 293 cells after 3 h treatment. **(b)** ROS production upon Cu@textile incubation. ROS was detected by 2,7-dichlorofluoresin diacetate (DCFH-DA). **(c)** SEM photographs of *P. aeruginosa* after treatment with Cu@textile for 30 min. Scale bars, 5 μm. **(d, e)** Colonies and quantitative c.f.u. count of *M. tuberculosis*, *P. aeruginosa* and *A. baumannii* after co-culturing with Cu@textile for 3h.

To evaluate the biocompatibility of Cu@textile to mammalian cells, the Cu@textile samples were co-cultured with human embryonic kidney (HEK) 293 cells to analyzed the cell viability (Qian et al., 2022; Yu et al., 2024). Negligible cytotoxicity were observed after Cu@textile treatment in comparison to untreated cells (Figure 2a). Copper ions are known effective catalyst of Fenton reaction, which can enhance reactive oxygen species (ROS) generation, thereby damaging bacterial structure and eliminating the bacteria (Kumar et al., 2020; Xia et al., 2023; Huang et al., 2024; Zhang et al., 2021; Liu et al., 2024). As a proof of concept, ROS production induced by Cu@textile at different treated time was investigated. Cu@textile exhibited greatly promoted ROS generation at various treatment times, revealing a 1,400-fold increase in ROS generation compared to cotton textile after a 30-min incubation (Figure 2b). However, when glutathione was added, the antibacterial activity completely reversed (Supplementary Figure S3). These data prove ROS generation was indispensable for Cu@textile to achieve bacteria killing. Owing to the high efficiency of ROS generation, Cu@textile treatment caused severe *P. aeruginosa* membranes shrinking and cytoderm collapse, indicating that Cu@textile possess excellent antibacterial efficacy (Figure 2c). In order to evaluate the antimicrobial capability of Cu@textile, three typical respiratory pathogens, including *P. aeruginosa*, *A. baumannii* and *M. tuberculosis*, were utilized to investigate by colony-forming unit assay. The results showed that Cu@textile effectively eliminate over 99% of *M. tuberculosis*, characterized by rigid cytoderm making it resistant to pharmaceutical agents. Even more remarkable was the complete eradication of *P. aeruginosa* and *A. baumannii*, achieving a 100% kill rate (Figures 2d,e). This outstanding performance underscored the Cu@textile’s exceptional ability to completely suppress the growth of these bacteria, suggesting that Cu@textile may serve as a potent antibacterial textile against the spread of various respiratory pathogens.

## 4 Conclusion

In summary, we developed a straightforward method for preparing antibacterial textiles by incorporating copper ions into cotton textiles at the molecular level. This approach ensured that copper ions coordinate with oxygen atoms in cellulose molecules, achieving even distribution throughout the Cu@textile. The strong induction of reactive oxygen species (ROS) within bacteria by copper ions resulted in robust bacterial cytoderm damage and broad-spectrum antibacterial efficacy against common respiratory bacteria, including *P. aeruginosa*, *A. baumannii* and *M. tuberculosis*. Consequently, Cu@textile may provide a robust measure for hospitals, capable of significantly reducing the risk of respiratory bacteria, even respiratory virus transmission, which in turn minimizes the occurrence of infections among inpatients.

## Data Availability

The original contributions presented in the study are included in the article/Supplementary Material, further inquiries can be directed to the corresponding authors.
