# Supplementary material for: Antibacterial Cu-doped cotton textile against respiratory pathogens for preventing hospital-acquired infections
Source: Front Bioeng Biotechnol. 2025 Sep 10;13:1641123. doi: 10.3389/fbioe.2025.1641123 (PMC12457280; doi:10.3389/fbioe.2025.1641123)
Supplement: Supplementary file 1 [file DataSheet1.pdf]

Supporting Information

Antibacterial Cu-doped cotton textile against respiratory pathogens for preventing hospital-acquired infections

Jianhui Gao, Deliang Liu, Zhiqiang Lin, Yang Zhou, Zhuojun He, Xiafei Dai, Pengfei Zhao*, Hongzhou Lu*, Mingbin Zheng*

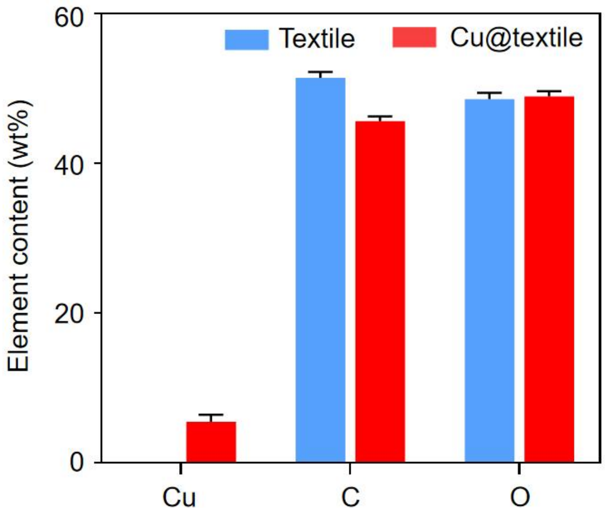

Figure S1. Element content of Cu, C and O on textile and Cu@textile surface.

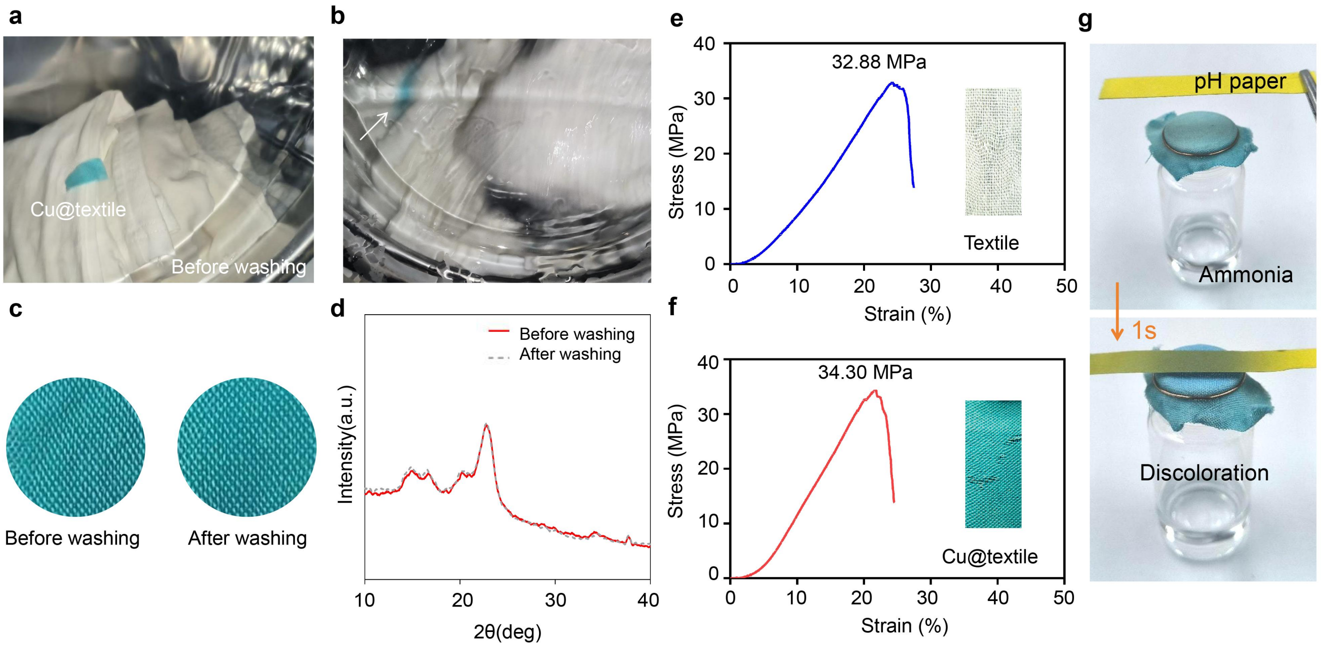

Figure S2. a, b) Photographs of the Cu@textile samples before and during washing. **c, d)** Photographs and XRD patterns of the Cu@textile samples before and after

washing. **e, f)** Tensile stress–strain curves of the textile and Cu@textile. **g)** The air permeability of the Cu@textile.

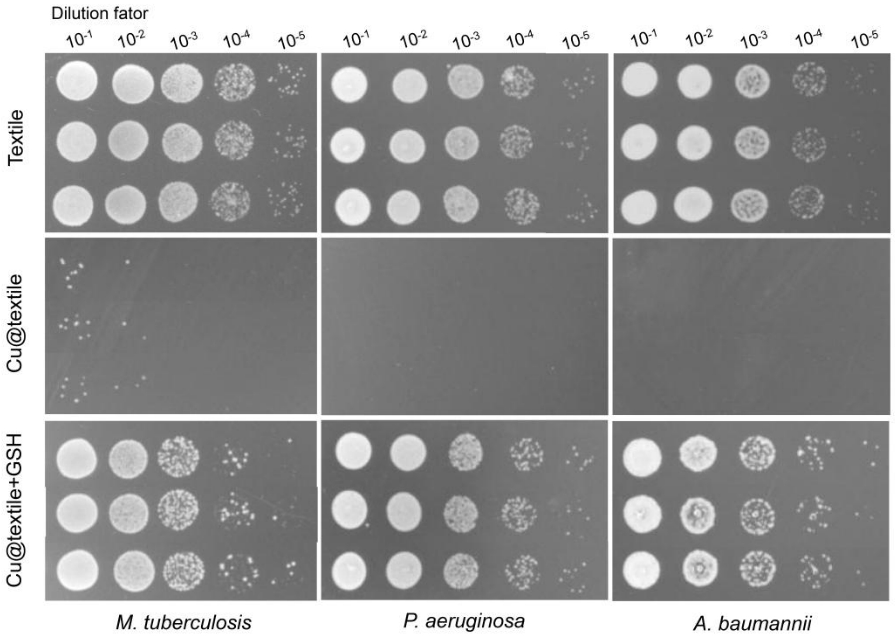

Figure S3. Colonies of *M. tuberculosis*, *P. aeruginosa* and *A. baumannii* after co-culturing with textile, Cu@textile and Cu@textile+GSH for 3h.
